# Prevalence of Different Etiologies of Excessive Gingival Display: Identifying Diagnostic Patterns

**DOI:** 10.1155/tswj/8869911

**Published:** 2026-02-26

**Authors:** Lubna Ahmad Amro, Mahetab Mohamed Abdalwahab, Nada Zazou, Ahmed Elsayed Hamed Amr

**Affiliations:** ^1^ Department of Oral Medicine, Periodontology and Oral Diagnosis, Faculty of Dentistry, October University for Modern Sciences and Arts, 6th of October City, Egypt, msa.eun.eg; ^2^ Department of Oral Medicine, Periodontology and Oral Diagnosis, Faculty of Dentistry, Ain Shams University, Cairo, Egypt, asu.edu.eg

**Keywords:** altered passive eruption, excessive gingival display, gummy smile, hyperactive lip, short lip

## Abstract

**Objectives:**

This study is aimed at evaluating the prevalence of etiologies of excessive gingival display (EGD) in Egyptian females including gingival enlargement (GE), altered passive eruption (APE), incisor over‐eruption (IO), protrusion (P), vertical maxillary excess (VME), short upper lip (SUL), and hyperactive upper lip (HUL) and to identify clinical diagnostic patterns.

**Methods:**

A total of 160 participants showing EGD > 2 mm were recruited. Clinical photos, videos, and measurements of facial proportions, upper lip length, upper lip mobility, incisor display upon rest, clinical crown dimensions, occlusal plane discrepancies, probing depth, transgingival probing, and keratinized gingiva were recorded and analyzed.

**Results:**

Mean age was (27.62 ± 6.21) years. Overall prevalence of EGD 13.3% among them 55.8% EGD caused by single etiology, 44.3% EGD caused by multiple etiologies. 29.4% APE, 16.3% SUL + APE, 10% VME + APE, 8.8% VME, 6.3% HUL, 5% Incisor over‐eruption, 3.8% GE, 3.8% SUL + GE, 3.1% VME + HUL, 2.5% SUL, 2.5% APE + HUL, 1.3% VME + GE, and 1.3% $\begin{array}{c} \text{VME}+\text{SUL}+\text{APE} \end{array}$…

**Conclusions:**

APE both alone and combined with another etiology is the most prevalent cause of EGD and the most common diagnostic pattern is APE + SUL among Egyptian females. Single‐factor and multifactorial EGD showed no significant difference in prevalence (*p* = 0.115), suggesting a similar likelihood of occurrence.

**Clinical Relevance:**

This study aimed to provide the clinician with a step‐by‐step guide for EGD comprehensive diagnosis, highlight the differences in prevalent etiologies between different populations and identify diagnostic patterns.

## 1. Introduction

Smiles are a pivotal method of communication that people generally link to success in many areas of life [1]. The amount of gingiva displayed in a person′s smile is considered a very important parameter in facial esthetics [2]. Some authors defined excessive gingival display (EGD) as exposure of more than 2 mm of gingiva when a person smiles [3]; others suggested more than 3 mm [4] or 4 mm [5, 6]. The prevalence of EGD is about 10% of the population, more in females [4, 7, 8, 9]. EGD can occur due to various intraoral or extraoral etiologies [10].

Intraoral etiologies include gingival enlargement (GE), incisor over‐eruption, and altered passive eruption (APE) [11, 12]. GE usually occurs due to gingivitis and/or drug‐induced gingival overgrowth (DIGO) [12]. Over‐eruption of the maxillary incisors causes a more coronal position of the gingival margin, causing EGD, associated with tooth wear at the anterior region or anterior deep bite [13]. Normal tooth eruption involves active eruption when the tooth moves out of its crypt, and passive eruption when the gingival margin migrates apically till it comes in proximity to the cementoenamel junction (CEJ) [2, 14, 15]. APE occurs when the gingiva fails to migrate apically during passive eruption, remaining in a position coronal to the CEJ, visible clinically as a short tooth [11, 12, 16]. APE is classified according to the amount of keratinized gingiva and the position of the osseous crest relative to the CEJ [15, 17, 18]. After much controversy regarding the age at which APE should be diagnosed, most authors agreed on around 18–19 years [18, 16, 19, 20, 21].

Extraoral etiologies that cause EGD include: vertical maxillary excess (VME), bimaxillary protrusion, short upper lip (SUL), and hyperactive upper lip (HUL) [22]. VME is excessive vertical growth of the maxilla [23]. Bimaxillary protrusion occurs when there are protrusive, proclined upper, and lower incisors combined with lip incompetence, EGD, and anterior open bite, commonly seen in Asian populations. [24, 25]. SUL occurs when upper lip length (ULL) measurement is less than 22 mm in males and 20 mm in females [26, 27]. Lip mobility is the amount of displacement that occurs to the lip from rest position during smiling; HUL is associated with hyperfunction of the lip elevator muscles [28]. Usually, a combination of two or more etiologies may be present, hence impeccable diagnosis is needed to formulate a suitable treatment plan [29].

The last evaluation of the Egyptian population found the prevalence of EGD to be 11.8% without specifying the etiology [30]. Little has been published about the prevalence of the various etiologies of EGD; however, the few existing studies show inconsistent data; some authors concluded that APE was the most common cause of EGD [11, 31]; others reported VME [23], but the most recent studies reported that APE and HUL were the most common [9, 32, 33]. This lack of population specific data limits clinicians′ ability to establish appropriate diagnosis and treatment plans. Moreover, most of the existing EGD data was collected from studies conducted on European patients and may not always apply to other populations. Fewer studies have been performed on different populations and have yielded different norms, measurements, and prevalences. This study is aimed at determining the prevalence of EGD among the Egyptian population, evaluating the distribution of its underlying etiologies, and assessing clinical diagnostic patterns using a simple, reproducible diagnostic outline.

### 1.1. Materials and Methods

#### 1.1.1. Sample Size Calculation

According to Elhiny [30], the prevalence of gummy smile is 11.8% among the Egyptian population; and based on the distribution of the Egyptian population from https://www.capmas.gov.eg/, a total sample size of 160 patients was sufficient to detect the effect size estimate 95% confidence interval and at a significance level of 5% (*p* < 0.05). The sample size was calculated according to G∗Power software Version 3.1.9.4. Where *f*S is the effect size, *α* = 0.05, *β* = 0.2 and Power = 1 − *β* = 0.95.

#### 1.1.2. Study Design

A total of 160 medically free (MF) female patients between 18 and 40 years with intact nonperiodontally affected maxillary anterior teeth who had more than 2 mm maxillary gingival display on maximum smiling were recruited for this cross‐sectional study from patients attending the outpatient clinic of the Oral Medicine, Periodontology and Oral Diagnosis Department, Faculty of Dentistry, Ain Shams University and Outpatient Clinic of the Oral Medicine and Diagnosis department, Faculty of Dentistry, MSA University from September 2022 till December 2023.

Although several definitions of EGD exist, the authors included EGD of more than 2 mm to avoid Neyman bias. Only healthy individuals were recruited to minimise any confounding due to DIGO or GE related to any disease or condition. The sample comprised only female patients because there is a significant gender difference in the prevalence of EGD that may influence the results [3, 7, 8, 32, 33]. Sample age was chosen with respect to the age at which APE is diagnosed [16] and the reported decrease in prevalence of EGD with age [34, 35] as well as the increase in prevalence of incisor overeruption with age [36], a known etiology of EGD; hence, the inclusion of older or younger age groups may confound results. No selection bias was identified, and the sample included females of different socioeconomic levels, education levels, geographic distribution, and age groups representative of the reference population.

This cross‐sectional study was conducted as per the guidelines of strengthening the reporting of observational studies in epidemiology (STROBE) [37]. The study was reviewed and approved by the Research Ethical Committee of the Faculty of Dentistry at Ain Shams University (Approval Number: FDASU‐Rec IM122107, approval date: 22/12/21). Written informed consent form was read, understood, and signed by all the participants. Clinical photographs and videos of patients included in this study were taken, and they were subjected to comprehensive oral examination [13]. The measurements in this study were all taken by the same calibrated periodontist using a University of North Carolina (UNC) Periodontal 15 mm probe (Zeffiro) and a digital caliper (Ingco) [38, 39, 40]. Upon completion of candidate recruitment, all recorded data were entered into a digital spreadsheet and analyzed.

### 1.2. Comprehensive Oral Examination

**Figure 1:**
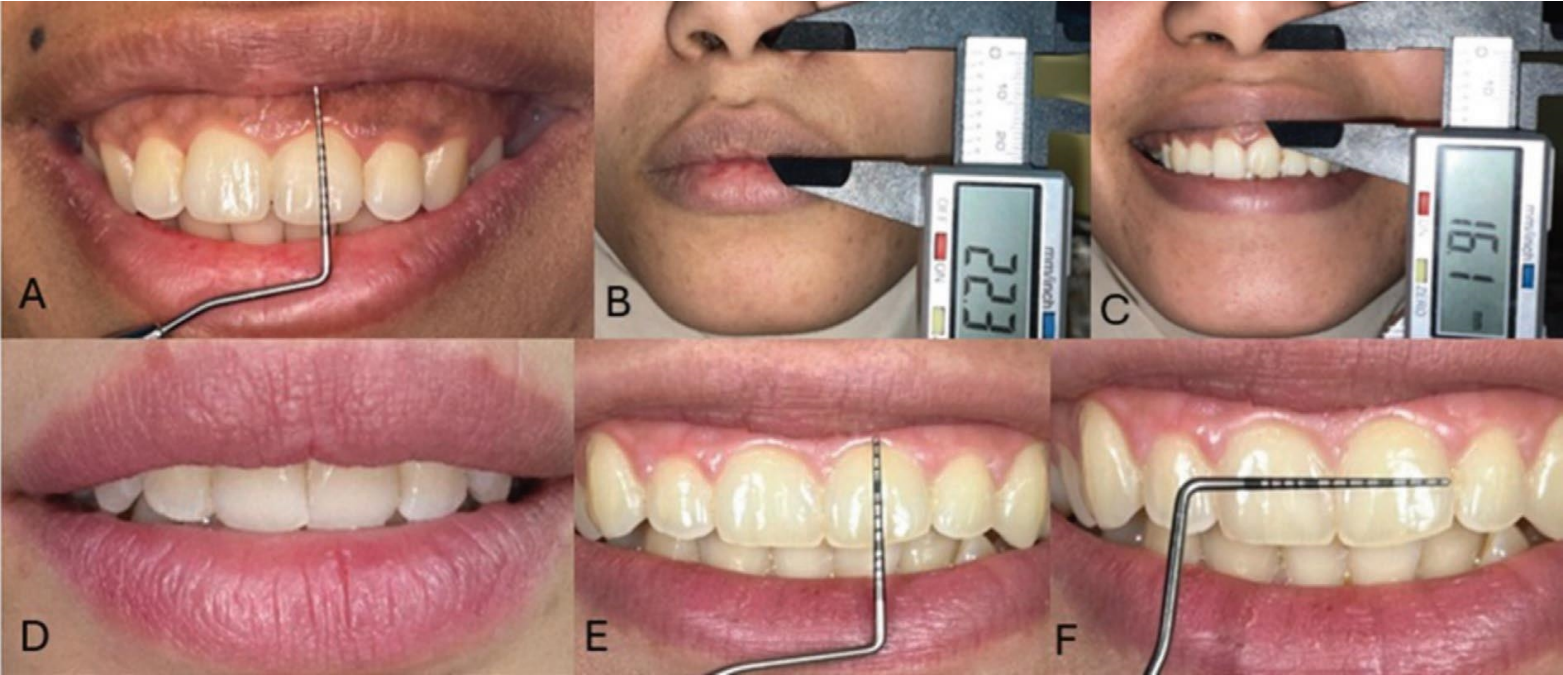
(A) Screening by measuring GD on maximum smile with UNC periodontal probe, (B) measuring ULL at rest using digital caliper, (C) measuring ULL on maximum smile using digital caliper, (D) clinical photo of a patient with incompetent lips and incisal display at rest, (E) measuring length of clinical crown of upper central incisor using UNC periodontal probe, (F) measuring width of clinical crown of upper central incisor using UNC periodontal probe.

**Figure 2:**
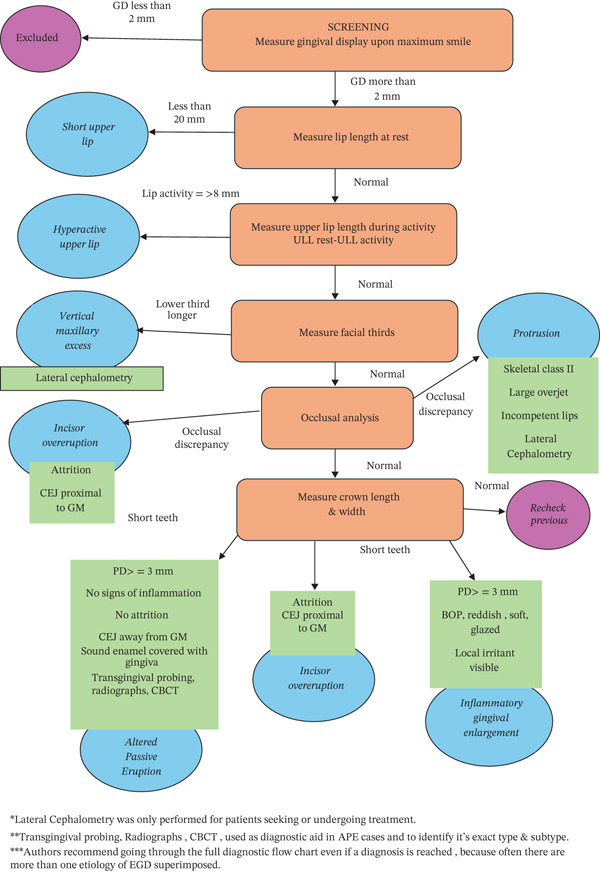
EGD comprehensive oral diagnostic flow chart.

Patients were asked to relax their lips without any muscle contraction; incisor display and ULL were measured; an incisor display of 2 mm at rest was considered normal. Afterward, static evaluation of ULL at rest was done by measuring the lip from the base of the nose (subnasale point) to the inferior part of the upper lip (upper lip stomion point). When the ULL reading was less than 20 mm, SUL was diagnosed [3, 26]. Dynamic smile evaluation measurement was based on complex smile [41]. Lip activity was calculated by measuring ULL during complex smiling using the same anatomical landmarks and subtracting it from the measurement of ULL at rest [13, 42]; resulting value greater than or equal to 8 mm was considered HUL [13, 33]. Evaluation of facial height was done by division of the face into three thirds; VME was diagnosed when the length of the lower third of the face was more than the length of the middle and upper thirds, respectively [13, 43]; only the patients seeking treatment were referred to do a lateral cephalometric radiograph for ethical reasons (Figures 1 and 2).

Occlusal analysis was done by evaluating interincisal relationship, incisor proclination, overjet, and overbite and by checking the harmony of the dental arches to identify that anterior and posterior segments are on the same plane and have no major discrepancies. Cases with positive measurements of overjet and/or overbite that were associated with proclined incisors, Class II occlusion, and incompetent lips (that were not SUL) were diagnosed as protrusion. Only those seeking treatment were referred to do lateral cephalometric radiographs to confirm [13, 41, 42, 43, 44, 45]. In cases where occlusal harmony was found paired with a positive measurement of the lower third of the face, the case was diagnosed as VME [13, 44]. (Figures 1 and 2).

Clinical crown length (CL) and crown width (CW) of the upper maxillary anteriors were measured and a tooth with CW:CL ratio less than 80% was considered a short tooth [46, 47]. Short teeth with clinically visible incisal attrition with or without deep bite, and CEJ detected in proximity with gingival margin upon probing were diagnosed as incisor overeruption. In addition, anterior occlusal plane at a different level than the posterior occlusal plane, the most probable diagnosis was incisor overeruption [11, 13, 48, 49, 50]. Short teeth with no incisal attrition, probing depth more than 3 mm without signs of inflammation, CEJ away from the gingival margin with clinically detectable part of anatomical crown enamel covered by gingiva was diagnosed APE. Keratinized attached gingiva was measured for all patients and it is of particular relevance when diagnosing the type of APE. Further investigations were done for only patients seeking treatment including transgingival probing, radiographs, and CBCT to confirm the diagnosis and classify the case [4, 16, 17, 18, 44]. Short teeth with probing depth more than 3 mm with bleeding on probing and signs of inflammation were considered of GE [5] (Figures 1 and 2).

### 1.3. Statistical Analysis

The mean and standard deviation values were calculated for quantitative data, whereas the frequencies were calculated for qualitative data. Fisher′s exact and chi‐square tests were used to determine the relationship between frequencies. The significance level was set at *p* ≤ 0.05. Statistical analysis was performed with IBM SPSS Statistics Version 20 for Windows.

## 2. Results

A total of 1455 adult female subjects attending the outpatient clinic were interviewed and screened by measuring the gingival display upon maximum smile; 194 subjects showed EGD > 2 mm and fit the other selection criteria, of whom 160 patients gave consent and agreed to undergo oral comprehensive diagnosis, clinical photos, and videos, hence were recruited. The study contained no illiterate patients; 80 patients were unemployed housewives or did not receive full education and 80 patients were employed or university students. The population consisted of residents from six governates: Cairo, Giza, Faiyum, Gharbia, Menofia, and Alexandria. The authors aimed to target different education levels and socioeconomic levels to avoid any potential bias. All charts were completed with full patient demographic data, measurements, and clinical photos/videos by a calibrated periodontist. Afterwards, charts were revised by a blinded calibrated periodontist. Patient contacts were taken, and if any missing or vague data were found, the patient was recalled. The prevalence of EGD was 13.3%. The mean age of the study population was 27.62 ± 6.21 years, with the youngest patient being 18 years old and the oldest patient being 40 years old. EGD was caused by single etiology in 89 patients (55.8%) and by combined etiologies in 71 patients (44.3%) with insignificant difference (*p* = 0.155) (Supporting Information 1–3).

Clinical parameters that were measured for every patient to reach a proper diagnosis were entered into a spreadsheet and revised by two blinded periodontists to ensure accuracy. The study contained binary and continuous variables. For continuous variables, the mean and standard deviation were calculated. The mean gingival display on maximum smile was (3.84 ± 1.13) mm. Mean incisor exposure during rest was (2.50 ± 1.67) mm. Mean ULL at rest was (20.54 ± 1.54) mm. The mean ULL during smiling was (15.23 ± 1.66) mm. Mean lip activity was (5.31 ± 1.75) mm. The mean CL was (8.48 ± 1.16) mm. Mean CW was (7.98 ± 0.72) mm. The mean attached keratinized gingiva width was (5.93 ± 1.71) mm. Binary variables were recorded as “present” or “absent” Increased lower third face measurement was found in 26.9% of the population. Attrition was visible clinically in 8.8% of the population. GE was present in 10.1% of the population. Increased overjet was seen in 3% of the population. After analysis of all the clinical measurements, photos, and videos, diagnosis was reached. When EGD was caused by more than one etiology superimposed together, the combination was treated as a separate diagnostic entity to help recognize clinical diagnostic patterns of combined causes that tend to recur together (Table 1) (Supporting Information 1–3). The presence of each etiology alone and combined with other etiologies was calculated from our primary results (Table 2) (Supporting Information 1–3).

**Table 1:** Diagnosed etiology or multifactorial diagnostic pattern of EGD as percentage and number of patients.

Diagnosis	Percentages	Number of patients
APE	29.40%	47
SUL combined with APE	16.30%	26
VME Combined with APE	10.00%	16
VME	8.80%	14
HUL	6.30%	10
Incisor overeruption	5.00%	8
SUL combined with GE	3.80%	6
Gingival enlargement	3.80%	6
VME combined with HUL	3.10%	5
APE combined with HUL	2.5%	4
SUL	2.5%	4
VME combined with SUL combined with APE	1.3%	2
VME combined with GE	1.3%	2
HUL combined with incisor overeruption	0.6%	1
SUL combined with APE and Incisor overeruption	0.6%	1
VME combined with incisor overeruption	0.6%	1
APE combined with GE	0.6%	1
VME combined with SUL	0.6%	1
GE combined with protrusion	0.6%	1
VME combined with SUL and protrusion	0.6%	1
VME combined with APE and protrusion	0.6%	1
APE and protrusion	0.6%	1
HUL and protrusion	0.6%	1

**Table 2:** Percentage, patient number, and *p* value of etiology when diagnosed alone and when diagnosed combined with other etiologies.

Etiology	Present alone(patient %)	Present alone pt number	Present combined with other etiology(Patient %)	Present combined pt Number	*p*
APE	29.40%	47	32.5%	52	0.615
SUL	2.5%	4	23.2%	37	< 0.001∗
VME	8.8%	14	18.1%	29	0.022∗
HUL	6.3%	10	6.8%	11	0.827
Incisor overeruption	5%	8	1.8%	3	0.132
GE	3.8%	6	6.3%	10	0.317
Protrusion	0%	0	3%	5	< 0.025∗

*Note:* Asterisks indicate statistical significance.

## 3. Discussion

This study evaluated the prevalence of intraoral and extraoral etiologies of EGD with identification of clinical diagnostic patterns in Egyptian females. The amount of gingival display considered EGD is controversial [3, 4, 5, 6, 47]. This current study reported a prevalence of EGD in Egyptian females 13.3%, which falls within the previously reported ranges: 10%, 11.8%, and 10.9% without etiological distinction [7, 33, 50] and more prevalence and extent in females [3, 9, 31, 33, 51, 52]. Despite more cases showing EGD caused by a single etiology, both single and multifactorial EGD occurred in comparable frequencies, stressing the importance of a full comprehensive oral diagnosis for every EGD patient to formulate a full treatment plan.

### 3.1. Single Etiology

According to our study, APE was the most common etiology of EGD, both alone and combined with another etiology; 29.4% and 32.5% respectively. The prevalence of APE alone was 32.2% in the Saudi population [53], 35% in the Spanish population [52], 20.8% in the North American population [39], and 47.3% in the Turkish population [4]. An older study reported the prevalence of APE in the South African population of only 12.1% [33]. This variation may reflect racial and ethnic differences in dentogingival morphology and diagnostic criteria.

In this current study, prevalence of SUL alone was quite low 2.5%, whereas much higher combined with other etiologies 23.2%; previous studies found prevalence of SUL alone was 2.7% in North American females [33], and 9% in the Turkish population [4]. Our results were similar to North American females but lower than the Turkish population because SUL was more common in males than in females [32]. These differences likely reflect gender distribution and ethnic variation in ULL.

The prevalence of VME alone was 8.8%, whereas the prevalence of VME combined with other etiology was 18.1%. In previous studies, the prevalence of VME was reported as 10% among young non‐EGD and EGD individuals with a higher prevalence of skeletal deformities in females [7, 54] and 31.4% in an Iranian sample who had various smile line problems [54]. These findings suggest that VME contributes consistently to EGD throughout populations with some variations related to skeletal patterns and sample characteristics.

The prevalence of hyperactive lip alone was 6.3% and combined was 6.8%. The prevalence of HUL alone in North American patients was 10.7% [33]. Turks showed prevalence was 87.3% in a study containing both genders, males outnumbering females [4]. Another study that divided individuals into racial groups found a statistically significant difference between races and genders, with males having a higher prevalence of hyperactive lip [33]. HUL is much more prevalent among the Turkish population and the male gender in particular; racial and dynamic lip muscle differences may explain this phenomenon.

Incisor over‐eruption, a relatively less common cause of EGD, was 5% alone and 1.8% combined with other etiologies. The prevalence of GE was 3.8% alone and 6.3% combined with other etiologies, and many of the diagnosed cases also reported mouth breathing, consistent with the association between mouth breathing and GE [55]. Protrusion was only found in combination with another etiology in 3% of the patients. These findings underline that although less frequent than APE or VME, occlusal and inflammatory etiologies can contribute to EGD and often may coexist with other etiologies.

### 3.2. Diagnostic Patterns

The most common diagnostic pattern in this study was SUL combined with APE at 16.3%; however, the prevalence in the Turkish population was only 1.8% [4]. However, but the turkish study contained both genders, males outnumbering females. Another prevalent diagnostic pattern found in 10% of the sample was VME combined with APE. To the authors′ knowledge prevalence of VME combined with APE has been reported before in case studies only [56, 57].

Another diagnostic pattern was SUL combined with GE with prevalence 3.8%, usually clinically observed with incompetent lips and mouth breathing [58]. With higher prevalence in females, SUL may contribute to lip incompetency [59], but more studies need to be done to investigate this phenomenon. Another common diagnostic pattern was VME combined with HUL present in 3.1% of the population. In the Iranian population, 15.3% had VME and HUL combined [54] and it was reported in several case reports [10]. VME and GE combined were found in 1.3% of the sample; the same pattern of VME combined with GE was reported in 10.9% of the Iranians, [54] the different value may be attributed to racial differences [33]. In this current study, prevalence of APE and combined HUL was 2.5%, also reported to be 22% in North Americans [33], and 40% in Turks [4]. These wide variations may be attributed to differences in lip dynamics, racial morphology, and sample composition. [33] Less frequent combinations such as VME combined with SUL and APE were also observed.

Among limitations of this study were that only healthy subjects were included to minimize confounding and only females were included because a statistically significant difference between genders was found in both upper lip parameters and prevalence of APE [9, 31, 33, 51, 52]. Another limitation was that only candidates with intact anterior teeth were included, which was important in the measurement of CL and CW. Another limitation was that lateral cephalometry and transgingival probing/radiograph/CBCT were only done for patients seeking treatment for ethical reasons. Had they been done for all patients, they would have provided valuable findings.

APE was a common cause of EGD in Egyptian, North American, Saudi, Turkish, and Iranian populations ranging from 20% to 48%; however, South Africans showed a lower prevalence of APE at 12% only. SUL was more common in the Egyptian population than in any other, very commonly occurring combined with APE. Iranians have shown a much higher prevalence of VME compared with other populations, possibly due to skeletal differences between races. Bimaxillary protrusion is very common in Caucasian populations. Hyperactive lip is very common in the Turkish population, with a prevalence more than 7 times that of other populations, likely due to gender distribution and muscle dynamics. Much variation exists between populations, and more research needs to be done on larger sample sizes to be able to pinpoint these differences. Further studies across diverse populations need to be done.

## 4. Conclusion

APE is the most prevalent cause of EGD both when diagnosed alone or when diagnosed combined with other etiologies. The most common diagnostic pattern is APE combined with SUL among Egyptian females. This current study proved that EGD may be equally likely to be caused by single or combined etiology (*p* = 0.155), highlighting the need for comprehensive oral examination to properly diagnose EGD. Within the limitations of this study, results can be applied to young Egyptian females; further studies should be done on male Egyptian subjects and female and male subjects from other populations to overcome variations in many of the parameters involved in the diagnosis of EGD. Further studies need to be done on the diagnostic patterns found in this study with a larger number of participants and on other populations show the same or different diagnostic patterns.

**Nomenclature**
STROBE: strengthening the reporting of observational studies in epidemiology
EGD: excessive gingival display
DIGO: drug‐induced gingival overgrowth
ULL: upper lip length
UNC‐15 Probe: university of north carolina probe 15 mm
W/H ratio: width to height ratio
SD: standard deviation
APE: altered passive eruption
HUL: hyperactive upper lip
VME: vertical maxillary excess
SUL: short upper lip
GE: gingival enlargement

## Author Contributions

L.A.A.: conception and design of the study, data acquisition and analysis, interpretation of data, manuscript draft and revision, personal accountability. and figures; M.M.A.: data acquisition, analysis, interpretation, drafted and revised manuscript, and personal accountability. N.Z.: conception and design of the study, data acquisition and analysis, interpretation of data, manuscript draft and revision, and personal accountability. A.E.H.A.: conception and design of the study, data acquisition and analysis, interpretation of data, manuscript draft and revision, and personal accountability.

## Funding

No funding was received for this manuscript.

## Disclosure

All authors have read and approved the final version of the manuscript. The corresponding author Dr. Lubna Ahmad Amro had full access to all of the data in this study and takes complete responsibility for the integrity of the data and the accuracy of the data analysis.

## Ethics Statement

The study protocol was approved by the Research Ethics Committee of the Faculty of Dentistry, Ain Shams University (FDASU‐REC‐IM122107). All procedures were conducted in accordance with the Declaration of Helsinki. This cross‐sectional study was conducted as per guidelines of strengthening the reporting of observational studies in epidemiology (STROBE**).**

## Consent

All participants were informed about the nature and objectives of the study, and written informed consent was obtained prior to enrollment.

## Conflicts of Interest

The authors declare no conflicts of interest.

## Supporting Information

Additional supporting information can be found online in the Supporting Information section.

## Supporting information


**Supporting Information 1** contains the raw data of the candidates involved in this study.

**Supporting Information 2** contains the raw statistical analysis charts and tables for multiple etiologies before tailoring them into the results.

**Supporting Information 3** contains the raw statistical analysis charts and tables descriptive statistics of single etiologies before tailoring them into the results.

## Data Availability

The datasets generated and/or analyzed during the current study are available from the corresponding author on reasonable request.
