# Supplementary material for: Prevalence of Different Etiologies of Excessive Gingival Display: Identifying Diagnostic Patterns
Source: ScientificWorldJournal. 2026 Feb 26;2026:8869911. doi: 10.1155/tswj/8869911 (PMC12936854; doi:10.1155/tswj/8869911)
Supplement: Supplementary file 2 — Supporting Information 2 contains the raw statistical analysis charts and tables for multiple etiologies before tailoring them into the results. [file TSWJ-2026-8869911-s002.docx]

APE

**Results**

**Multinomial Test**

| *Multinomial Test* | | | |
| --- | --- | --- | --- |
|  | χ² | df | p |
| Multinomial | 0.253 | 1 | .615 |
|  | | | |

SUL

**Results**

**Multinomial Test**

| *Multinomial Test* | | | |
| --- | --- | --- | --- |
|  | χ² | df | p |
| Multinomial | 26.56 | 1 | < .001 |
|  | | | |

VME

**Results**

**Multinomial Test**

| *Multinomial Test* | | | |
| --- | --- | --- | --- |
|  | χ² | df | p |
| Multinomial | 5.233 | 1 | .022 |
|  | | | |

HUL

**Results**

**Multinomial Test**

| *Multinomial Test* | | | |
| --- | --- | --- | --- |
|  | χ² | df | p |
| Multinomial | 0.048 | 1 | .827 |
|  | | | |

Incisor over eruption

**Results**

**Multinomial Test**

| *Multinomial Test* | | | |
| --- | --- | --- | --- |
|  | χ² | df | p |
| Multinomial | 2.273 | 1 | .132 |
|  | | | |

GE

**Results**

**Multinomial Test**

| *Multinomial Test* | | | |
| --- | --- | --- | --- |
|  | χ² | df | p |
| Multinomial | 1.000 | 1 | .317 |
|  | | | |

Protrusion

**Results**

**Multinomial Test**

| *Multinomial Test* | | | |
| --- | --- | --- | --- |
|  | χ² | df | p |
| Multinomial | 5.000 | 1 | .025 |
|  | | | |
| *Note.*  Chi-squared approximation may be incorrect | | | |

Total

**Results**

**Multinomial Test**

| *Multinomial Test* | | | |
| --- | --- | --- | --- |
|  | χ² | df | p |
| Multinomial | 2.025 | 1 | .155 |
|  | | | |
