# Supplementary material for: Prevalence of Different Etiologies of Excessive Gingival Display: Identifying Diagnostic Patterns
Source: ScientificWorldJournal. 2026 Feb 26;2026:8869911. doi: 10.1155/tswj/8869911 (PMC12936854; doi:10.1155/tswj/8869911)
Supplement: Supplementary file 3 — Supporting Information 3 contains the raw statistical analysis charts and tables descriptive statistics of single etiologies before tailoring them into the results. [file TSWJ-2026-8869911-s003.docx]

Descriptives of measurements

| **Descriptive Statistics** | | | | | |
| --- | --- | --- | --- | --- | --- |
|  | N | Minimum | Maximum | Mean | Std. Deviation |
| Gingival display on maximum smile | 160 | 2.00 | 7.00 | 3.8375 | 1.12511 |
| Incisor exposure during rest | 160 | .00 | 9.00 | 2.5031 | 1.66871 |
| Lip length at rest | 160 | 15.00 | 25.00 | 20.5375 | 1.53937 |
| Lip length during smiling | 160 | 11.00 | 20.00 | 15.2281 | 1.66054 |
| Lip activity | 160 | 2.00 | 10.00 | 5.3094 | 1.75179 |
| Crown length | 160 | 6.00 | 13.00 | 8.4750 | 1.15851 |
| Crown Width | 160 | 6.00 | 10.00 | 7.9750 | .71551 |
| Keratinized gingiva | 160 | 3.00 | 11.00 | 5.9250 | 1.71398 |
| Valid N (listwise) | 160 |  |  |  |  |
